# Pure Cold-Induced Cholinergic Urticaria in a Pediatric Patient

**DOI:** 10.1155/2016/7425601

**Published:** 2016-11-29

**Authors:** Tina Abraham, David P. McGarry, John Frith, Jason Casselman, Haig Tcheurekdjian, Robert Hostoffer

**Affiliations:** ^1^Department of Allergy and Immunology, University Hospitals, Regional Hospitals, Richmond Heights, OH, USA; ^2^Department of Pediatrics, Akron Children's Hospital, Akron, OH, USA; ^3^Allergy/Immunology Associates, Inc., Mayfield Heights, OH, USA

## Abstract

Cold urticaria and cholinergic urticaria are two distinct entities. The presentation of exclusive cold-induced cholinergic urticaria is very rare. The patient described herein had experienced urticaria in the exclusive setting of exercising in a cold environment. Urticarial testing including laboratory and in-office testing was all negative. The patient has prevented urticaria symptoms with oral antihistamine therapy. Pure cold-induced cholinergic urticaria is rarely described in literature. This form of urticaria has yet to be described in a pediatric patient.

## 1. Case Report

Cold urticaria and cholinergic urticaria have been clearly defined as two separate entities. Review of literature shows that the cooccurrence of both as one distinct clinical feature is rare. While there have been a few reported cases of isolated cold-induced urticaria with cholinergic features in adults, we present the first case of pure cold-induced cholinergic urticaria in a pediatric patient.

A 16-year-old female presented with complaints of urticaria for the past 18 months. She developed the urticaria and pruritus exclusively in the setting of exercising in a cold environment. Urticaria was never triggered with only cold exposure or exercise in a warm environment. There was no significant contributing family history of disease. She also denied any other associated symptoms such as swelling, difficulty in breathing, dizziness, or any systemic symptoms. The patient's skin lesions were described as erythematous, pinpoint papules which covered the entire body but spared the face.

An ice cube test was performed and was negative. All laboratory findings of a complete blood count, basic metabolic panel, tryptase, C-reactive protein, aspartate aminotransferase (AST), alanine aminotransferase (ALT), bilirubin, alkaline phosphatase, chronic urticarial index, and thyroid panel were within normal limits. The patient was initiated on oral hydroxyzine therapy for cold-induced cholinergic urticaria and saw success with symptomatic improvement.

The exact mechanisms of both cold and cholinergic urticaria are unclear. The first report of a patient with cold urticaria was described in 1866 [1]. Cold urticaria is believed to be associated skin antigens manifested during cold exposure which are detected by IgE antibodies leading to mast cell activation and histamine release [2]. Cholinergic urticaria was first described in 1924 [3]. A suggested mechanism of cholinergic urticaria includes an allergic response to sweat antigens [2]. However, it is also seen in a patient with anhidrosis [4].

In cold-induced cholinergic urticaria, Ormerod et al. [5] proposes a common circulating factor, like IgE, leading to urticaria. Kaplan and Garofalo [6] first described cold-induced cholinergic urticaria in 1981 in four patients who clinically demonstrated cholinergic appearing urticaria caused by cold exposure. These patients showed morphological cholinergic urticaria in the setting of negative ice cube testing. Only one of these patients, an adult, had a similar history to our patient in that urticaria presented with exercising only in a cold environment. Geller [7] described a 9-year-old patient with cholinergic appearing urticaria with cold exposure but was not induced with physical activity. In a study of 220 patients with a history of cold urticaria, fifteen concomitantly had cholinergic urticaria as determined by positive ice cube and methacholine testing [8]. Two had negative ice cube tests but positive methacholine tests and were thus called cold-induced cholinergic urticaria. Ormerod et al. [5] described thirteen patients with cold-induced cholinergic urticaria defined by testing. Each had cholinergic urticaria from sweat provoking stimuli and then morphologically cholinergic urticaria in the setting of cold contact or cold exposure. Other patients have been described who develop cholinergic and cold urticaria but are not exclusively seen together [2]. Our patient is unique in that the urticaria is only seen with exercise in the cold.

Outside of preventing precipitating factors, treatment of cold-induced cholinergic urticaria should initiate with antihistamine therapy. In our case and other descriptions of the disease, antihistamines were used and have generally been successful. In the setting of antihistamine failure, omalizumab has been suggested.

The clinical discernment of urticarial disease requires a strict history collection strategy in order to remove triggers accurately. To our knowledge, this is the first pediatric patient with pure cold-induced cholinergic urticaria.
